# Impact of *Salvadora persica* aqueous extract on follicular development in female rats

**DOI:** 10.1186/s40659-026-00711-z

**Published:** 2026-06-22

**Authors:** Sabreen M. Ghareeb, Mahmoud Abd-Elkareem, Ahmed Abou-Elmagd

**Affiliations:** Department of Cell and Tissues, Faculty of Veterinary Medicine, Assiut University, Assiut, 71526 Egypt

**Keywords:** Miswak (*Salvadora persica*), Ovary, Follicles, Atresia, Rats

## Abstract

*Salvadora persica* (miswak) has many useful biological activities. Some studies have reported that miswak can be used as a contraceptive agent. However, no available investigations explain the histomorphological structure of ovarian follicles after miswak aqueous extract (MAE) administration. Twelve female Wistar albino rats were divided into two equal groups. In the control group (CG), the animals received normal saline daily for 4 weeks. While in the miswak treated group (MTG), the animals received orally 900 mg/kg of body weight of the MAE daily for the same period. At the end of the experiment, the rats were anesthetized and then euthanized by cervical dislocation. The ovaries were dissected, removed, weighted, fixed, and processed for histological examination by light and electron microscopy.

Results revealed that the ovaries of CG showed various stages of follicular development. While in the MTG, the ovaries exhibited follicular atresia. The immunoexpression of caspase-3, progesterone receptors (PR), and estrogen receptors alpha (ERA) in the MTG and CG were reported.

Our preliminary animal research indicates that MAE can inhibit follicular development and induce follicular atresia. These findings suggest a potential antifollicular and/or antiovulatory effect that requires confirmation through dose-response, fertility, toxicity, and clinical studies.

## Background

Follicular development is divided into two phases. The first, pre-antral phase, involves the formation, activation, and growth of primordial follicles. Oogonia develop from primordial germ cells. They differentiate into oocytes in the ovary. Each oocyte becomes surrounded by a single layer of flattened follicular cells, forming primordial follicle. Primordial follicle activation leads to the formation of the primary follicle. Primary follicle is formed of an oocyte surrounded by a single layer of cuboidal follicular cells. The secondary follicle has two or more layers of granulosa cells and a small number of theca cells. The second, antral phase is characterized by the formation of small and large antral (tertiary) follicles (antrum-filled follicular fluid cavity). Follicle growth continues through the phases of recruitment, selection, dominance, and preovulatory stage of follicular waves [1, 2].

Atresia is a degenerative process that occurs at all stages of follicular development from early ovarian formation onward [3, 4]. Granulosa cells adjacent to the basement membrane exhibit signs of pyknosis in early stage of atresia, while in the advanced stage of atresia, granulosa layer is no longer distinguishable, and macrophages are visible in the antrum [5].

Theca cells produce androgen that changes into estrogen in granulosa cells by aromatase enzyme and under effect of FSH. This estrogen enhances the sensitivity of granulosa cells to FSH. Follicles exposed to insufficient FSH during certain periods of development exhibit degeneration due to androgen accumulation in granulosa cells leading to apoptosis and atresia [5, 6]. Estrogens enhance follicle viability by inhibiting apoptotic DNA fragmentation [7]. In early stages of follicular development, atresia is triggered by oocyte apoptosis followed by the death of granulosa cells. However, in late pre-antral, antral and preovulatory follicles, atresia is stimulated by granulosa apoptotic cell death. Cell death in granulosa cells involves nuclear and cytoplasmic condensation, followed by the formation of apoptotic bodies [8].

The *Salvadora persica* tree is a member of the Salvadorceae family. It is a popular natural toothbrush sticks that were used centuries ago in dental hygiene by Muslims in India, Arabia, and Africa, especially in the Middle East [9]. Toothbrushes were constructed from roots and small branches (about 3 to 5 mm in diameter) of the plant [10]. Its synonyms in different countries include; miswak, Meswak, Siwak, sewak, Arak, Peelu Chewing Stick and is recommended by the World Health Organization to improve dental health and to promote oral hygiene. With proper training, the use of miswak has the potential to contribute positively to gingival health [11, 12]. It has been used in several medicinal and research activities as it is useful to produce antibacterial, antimycotic, antiplaque, analgesic, astringent, anticonvulsant, cytotoxic, antifertility, deobstruent, carminative, diuretic, and used in biliousness, and rheumatism [13]. Its scientific classification as follow:

- Kingdom: Plantae
- Division: Magnoliphyta
- Class: Magnoliopsida
- Order: Brassicales
- Family: Salvadoraceae
- Genus: *Salvadora*
- Species: *S. persica*

MAE has more pronounced activity than alcoholic extracts (methanolic/ethanolic) [10]. MAE contains important phytochemicals such as vitamin C, salvadorine, salvadourea, alkaloids, trimethylamine, cyanogenic glycosides, tannins, saponins and salts mostly as chlorides, sulfur, terpenes, 1-triacantanol, β-sitosterol, fluoride, silica, and flavonoids. Also, miswak stem possesses octacosanol and β-sitosterol-3-O-β-D-glucopyranoside [12–15].

Phytoestrogens are naturally occurring phytochemicals which are found in plants and plant products. They are structurally and functionally like 17-β-estrogen (isoflavones) or synthetic estrogens such as diethylstilbestrol [16].

MAE has an antifertility action on the ovary of female albino Wistar rats. It significantly reduces the ovarian weight [17]. It also reduces estrogen and increases progesterone levels and is safe to use as a natural contraceptive agent [18]. Other investigations indicated that miswak has an adverse effect on male and female reproductive system and fertility [19].

Hormonal contraceptives cause decreases ovarian size and suppress follicular growth and ovulation [20]. Contraceptive Depo-Provera (DMPA) causes follicular atresia with degeneration of growing follicles. While the absence of mature follicles and recent corpora lutea with Noristerat (NET-EN) treatment reflected the blockade of ovulation [21]. Ovarian morphology after prolonged use of steroid contraceptive agent showed high incidence of atretic follicles with degeneration in granulosa layer [22].

Research concerning the effect of MAE on the reproductive performance of females is scanty. Also, there is little available information about the effect of MAE on the histomorphology of developing and atretic ovarian follicles. Therefore, this study aimed to evaluate the effect of MAE on follicular dynamics and the possible use of MAE as a safe and effective contraceptive agent.

## Materials and methods

### Chemicals

The following chemicals were used: miswak extract, Saline solution, Formaldehyde, Glutaraldehyde, Osmium tetraoxide and chemicals for histological and immunohistological staining.

### Instruments

The following instruments were used: Micropipettes, Centrifuge, Shaker, Gastric tube, and Rotary evaporator.

### Preparation of miswak extract and dose

**Fig. 1:**
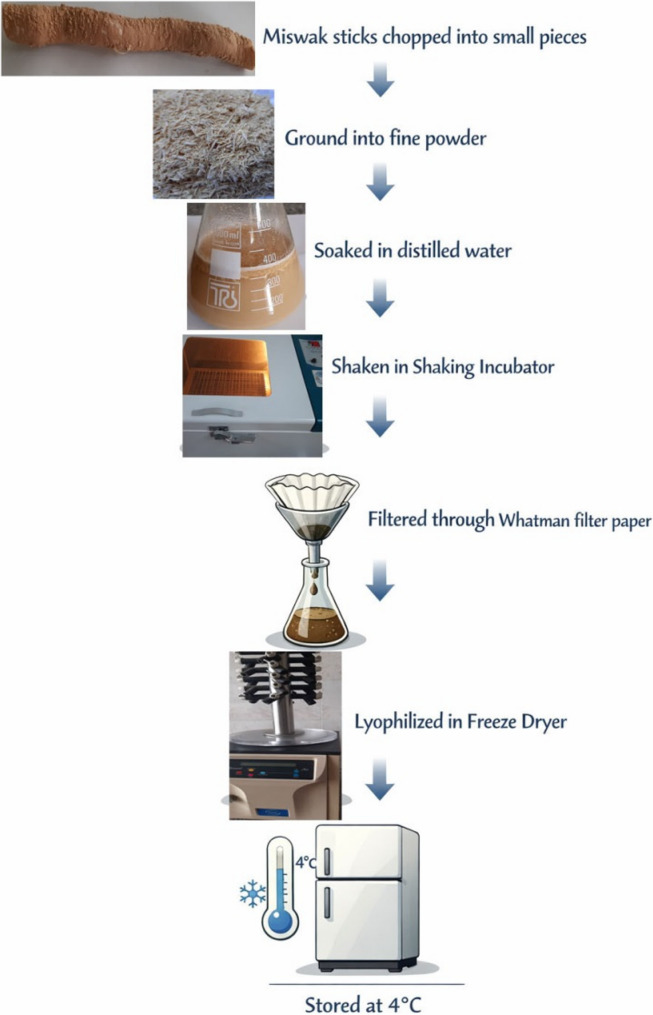
Illustrates the preparation of MAE

1. Raw miswak root sticks were purchased from authenticated company (Tahoor Company, Cairo, Egypt) with a trademark number, and they were free from additives.
2. Raw miswak root sticks were cut into pieces and let dry at room temperature for a week.
3. Then these pieces were ground to fine powder that soaked into distilled water in a sterile dry screw-capped bottle for 2 days at a temperature of 4 °C.
4. After that, this mixture of miswak was shaken in a shaking incubator (model JSSI-100 C) at 200 rotations/ min for 5 h at a temperature of 25 °C in the Central Laboratory of the Faculty of Veterinary Medicine, Assiut University.
5. The mixture is then filtered using Whatman filter paper till it obtains a clear solution.
6. The solution was lyophilized in a vertical freeze drier (model #6KBTES-55) in the Faculty of Science, Assiut University.
7. The extracts of miswak were stored at 4 °C until animal administration. These steps were illustrated in Fig. 1.

The animals were received orally 900 mg/kg of body weight of the MAE daily for 4 weeks [23].

### Chemical analysis of MAE

Chemical analysis of miswak extract was done in analytical chemical unit in Faculty of Science, Assiut University by using GC-MS (7890 A-5975B), USA, as follow:

1. Weigh 1gm of samples.
2. Add 1 ml of chloroform.
3. Sonication for 15 min at room temperature. Sonication is the act of applying sound energy to agitate particles in a sample, for the extraction of multiple compounds from plants.
4. Centrifuge at 10,000 rpm at 4 °C for 15 min.
5. Withdraw the clear organic layer and inject it into the Gas chromatographic-mass spectrometric **(**GC-MS) [10].

### Experimental design

Twelve female Wistar albino rats with an average weight of about (165.3 ± 3.269 g) were used. The rats were housed in cages and given free access to food and water. Animals were acclimatized for 7 days prior to experimentation. Prior to the beginning and throughout the experiment, the rats were housed at 24 °C room temperature and 12-hour light: 12-hour dark cycle. The rats were divided randomly into two main groups, CG and MTG, each containing 6 animals.

The experimental protocol was approved by the Local Ethical Committee and by the Institutional Review Board of the Faculty of Veterinary Medicine, Assiut University (Approval Number: 06/2023/0080) and was carried out in accordance with relevant guidelines and regulations. This research was done in compliance with the ARRIVE guidelines and regulations (https://arriveguidelines.org).

### Collection of samples

#### Vaginal smear/cytology

Vaginal wash (lavage) was performed according to [24], with the following steps:

1. Vaginal cells were collected by gently introducing a small amount of distilled water or saline into the vaginal canal using a sterile plastic pipette.
2. The liquid was slowly released, then drawn back into the pipette. This washing procedure was repeated 4 to 5 times using the same sterile pipette.
3. The resulting fluid contained a cell suspension (a few drops in volume).
4. A drop of this suspension was placed onto a glass slide, air-dried, and stained with methylene blue.

### Collection of blood samples and preparation of serum

- At the end of the experiment, blood samples were drawn from the retro-orbital venous plexus [25] of all rats in the two groups (6 from each group).
- Blood samples were left to coagulate at room temperature and then centrifuged at 3000 rpm for 20 min; the clear non-hemolyzed supernatant serum was quickly aspirated and kept at -20 °C till use.

### Collection of ovary samples

At the end of the experiment, the rats were anesthetized by using ketamine–xylazine. This was achieved by a dose of 87 mg ketamine/kg of body weight and 13 mg xylazine/kg of body weight intramuscular [26] and then euthanized by cervical dislocation. The ovaries were dissected, removed, weighted, fixed in neutral buffered formalin, and processed for embedding in paraffin.

### Paraffin sections

Samples were fixed in neutral buffered formalin and processed for paraffin embedding. Fixed tissue samples were dehydrated in ascending grades of alcohol, and then they were cleared in methyl benzoate and embedded in paraffin wax. Serial sections were cut at 5 μm by a Reichert microtome (Leica RM 2155, Germany) and mounted on glass slides. Sections were kept in an incubator at 40 °C for dryness. Then stained by Hematoxylin and eosin (Hx and E) for general histological examinations [27] and Periodic acid-Schiff’s (PAS) reaction for demonstration of neutral mucopolysaccharides [28]. All stained sections were examined under a light microscope (Olympus, USA), and photos were taken by an Olympus DP72 camera adapted into the microscope.

### Semithin and ultrathin sections

Approximately 2 mm fragments of ovaries were fixed in Karnovsky fixative at 4 °C for 24 h. The fixed specimens were washed several times in phosphate buffer, then post-fixed in 1% osmium tetroxide and dehydrated in ascending grades of alcohol and then embedded in epoxy resin. Semithin Sect. (1 μm) were stained with 1% Toluidine Blue [29]. The ultrathin Sect. (70 nm) were stained with uranyl acetate and lead citrate [30] and examined by a JEOL 100CX II transmission electron microscope (TEM) (JEOL, Tokyo, Japan) at the Electron Microscopy Unit of Assiut University.

### Preparation of digitally colored EM images

To enhance the visual contrast among various structures and cells in the same electron micrograph, we have digitally colored specific cells, such as the granulosa cells using Adobe Photoshop software version CS6 [31, 32]. We first open the image using Adobe Photoshop CS6 and then use any selection tool to pick the desired cell. Next, we select Adjustment from the image list, and then select Color Balance to set the desired color.

### Immunohistochemistry

- Neutral buffered formalin fixed samples from the ovary were processed and embedded in paraffin. 5 μm sections were prepared and processed for immunohistochemistry:
  1. To study the effect of MAE on expression of progesterone receptors, Progesterone Receptor Polyclonal Antibody (Bioss antibodies) was used.
  2. To study the effect of MAE on expression of estrogen receptors alpha, Estrogen Receptor Alpha Polyclonal Antibody (Bioss antibodies) was used.
  3. To study the effect of MAE on apoptosis, rabbit polyclonal antibody against caspase-3 (Catalog No.: A11953), AB clonal, USA was used.
- Additionally, poly Q stain 2 step detection system goat anti-mouse/rabbit HRP, peroxidase quench, DAB kit, Quartett, Germany was used.
- The immunohistochemical protocol used was according to the company instructions and previous reported studies [33–35] as follow:
  - The paraffin sections of ovary from both groups were treated with 3% H2O2 in H2O for 30 min at room temperature (RT) for blocking the endogenous peroxidase activity. The sections were washed with phosphate- buffered saline (PBS) (3 times × 5 min). After rinsing with distilled water, antigen retrieval was performed by incubating the sections with citrate buffer (pH 6.0) and placed in a microwave until boiling. After rinsing with PBS, the sections were blocked with 10% normal donkey serum (NDS) + 0.2% Triton-X100/PBS for 2 h at RT. Then, the sections were incubated overnight at 4 °C with primary antibodies.
- The sections were rinsed for (3 × 5 min) with PBS at pH 7.4 then incubated with secondary antibody for 30 min at room temperature. Then the sections were incubated for 5–10 min at room temperature with 3,3′-diaminobenzidine (DAB) and substrate-chromogen that produced a brown color at the antigen site. The slides were then counterstained with Harris hematoxylin for 30 s. The sections were dehydrated with ethanol alcohol 90%, then 100%, cleared in xylene, and covered using DPX.

### Hormonal analysis

Serum samples of all rats in the two groups (6 from each group) were processed for determination of the serum levels of FSH, LH, and estrogen concentration according to the company instructions and previous work [36, 37] as follow:

1. Bring all reagents to room temperature.
2. Place the desired numbers of coated strips into the holder.
3. Dispense standards and specimens into appropriate wells.
4. Add FSH, LH, or estrogen reagent to all wells.
5. Swirl the micropipette gently for 20–30 s to mix then cover.
6. Incubate 60 min at room temperature.
7. Remove the liquid from all wells; blot the plate with absorbent paper.
8. Wash three times by washing buffer, blot with absorbent paper.
9. Add working substrate solution.
10. Incubate at room temperature for 15 min.
11. Add stop solution to each well and gently mix.
12. Read the absorbance in each well at 450 nm within thirty min of adding a stop solution.

**Fig. 2:**
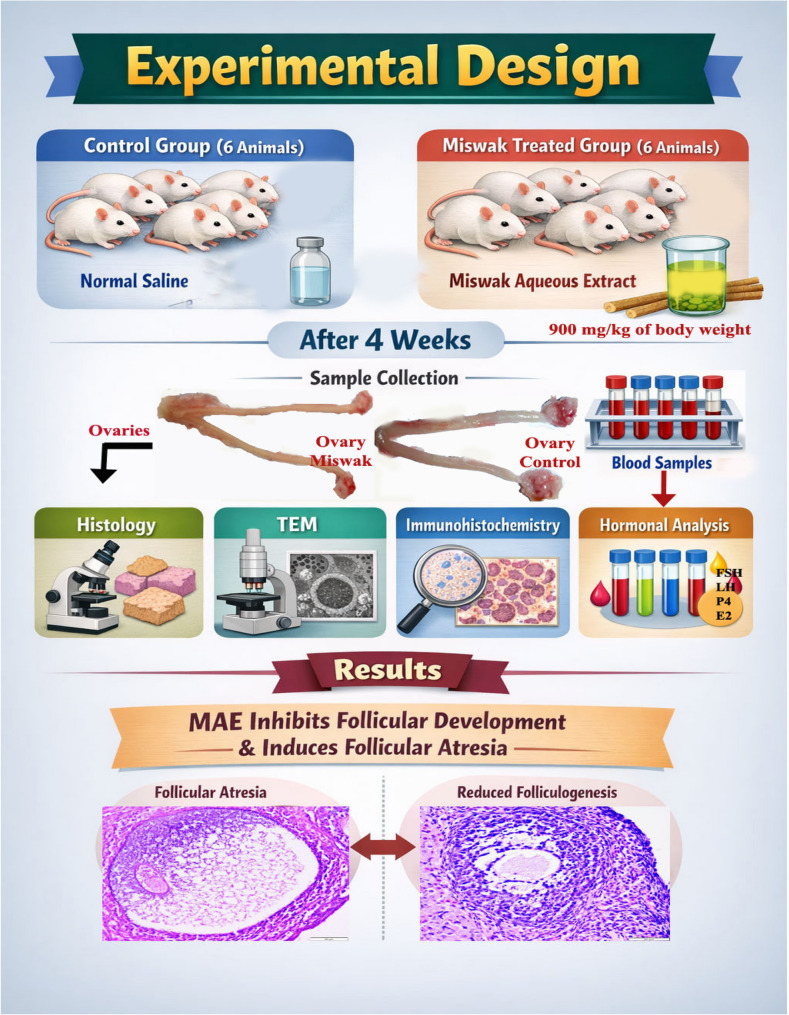
Summarizes the experimental design, the treatment protocol and study result

We summarize the experimental design, the treatment protocol, and study results in Fig. 2.

### Morphometric analysis and quantification of immunostaining markers

Morphometric analysis was performed on ovarian tissue sections obtained from 4 rats per experimental group. From each rat, 3 non-overlapping, randomly selected sections were analyzed. For each section, the numbers of primordial, growing, atretic antral, and mature follicles were counted. Section-level data were averaged per rat and the group comparison was performed using animal-level means. The mean number of each follicle type per group was then calculated. For quantification of immunostaining markers (caspase-3, ERA, and PR), the percentage of positively stained cells for each marker was measured using FIJI (ImageJ) in standardized fields per section, and the mean value was calculated per group.

Although each experimental group had six rats, only four animals were used for morphometric studies. The four animals were chosen based on optimal tissue preservation and processing quality. Based on pilot research impact sizes, this sample size was still sufficiently powered to detect significant differences.

### Statistical analysis

Data were presented as the mean ± SEM. Statistical analysis was performed using unpaired Student’s t-test [38], to compare MTG with the untreated control group. We used the Mann-Whitney U test (non-parametric) to compare mature follicle counts between the two groups, as the data were not normally distributed. Differences were considered statistically significant at (*p* < 0.05). *P* > 0.05 was considered not statistically significant. Statistical analysis was performed using GraphPad Prism 6.05.

### Ethical statement

The experimental protocol was approved by the Local Ethical Committee and by the Institutional Review Board of Faculty of Veterinary Medicine, Assiut University (Approval Number: 06/2023/0080) and was carried out in accordance with relevant guidelines and regulations. The animal handling procedures were performed in accordance with institutional animal welfare standards and that all efforts were made to minimize pain and distress. This research was done in compliance with the ARRIVE guidelines and regulations (https://arriveguidelines.org).

## Results

### Effect of the MAE on the hormonal levels

Blood serum analysis showed a non-significant decrease in the levels of FSH, LH, and estrogen hormones in the MTG group compared with the CG group (Table 1).

**Table 1:** Showing the effect of MAE administration on the serum levels of FSH, LH and estrogen in MTG compared with those in CG

Groups	CG	MTG
FSH mIU/mL	13.66 ± 0.835^a^	11.76 ± 0.902^a^
LH mIU/mL	7.68 ± 1.054^a^	5.68 ± 0.794^a^
Estrogen pg/mL	22.8 ± 1.040^a^	19.8 ± 1.348^a^

Values (Means ± SE) with the same superscripts (a) in the same row are not significantly different

### Effect of the MAE on the vaginal cytology

**Fig. 3:**
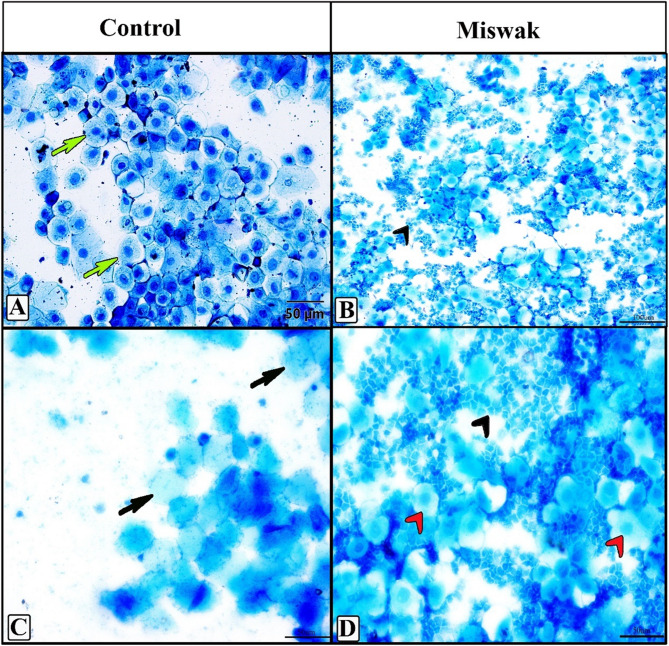
Vaginal smear stained by methylene blue showing the phases of the estrous cycle. **A**: Proestrus in CG showing numerous rounded nucleated cells (arrows). **B**: Persistent diestrus in MTG showing polymorphonuclear leukocytes (black arrowhead). **C**: Estrous in CG showing clumps of non-nucleated cornified epithelial cells free of nuclei (black arrow). **D**: Higher magnification of Persistent diestrus in MTG showing polymorphonuclear leukocytes (black arrowhead) and epithelial cells (red arrowhead). Scale bar B: 100 μm, A, C, and D: 50 μm

Vaginal wash examination revealed that female rats in the control group (CG) exhibited various stages of the estrous cycle. Most of them were in estrus, characterized by the presence of non-nucleated cornified cells that clump together. Some rats were in proestrus, characterized by the presence of numerous rounded or oval nucleated epithelial cells in the vaginal contents. These cells were uniform in size and appearance. In contrast, the vaginal wash of the MTG contained polymorphonuclear leukocytes and epithelial cells, indicating the diestrus phase of the cycle (Fig. 3; Table 2).

**Table 2:** Showing the effect of MAE administration on the estrus cycle

Groups	CG	MTG
♣ No. of animals in proestrus	1	0
♣ No. of animals in estrous	3	1
♣ No. of animals in metestrus	1	0
♣ No. of animals in diestrus	1	5

Nearly all female rats of MTG demonstrated diestrus phase. While most female rats of CG displayed the vaginal content of estrus phase, while the other animals of CG showed the other phases of estrus cycle

### Effect of the MAE on the follicular development and atresia

#### Primordial, primary, and secondary follicles

**Fig. 4:**
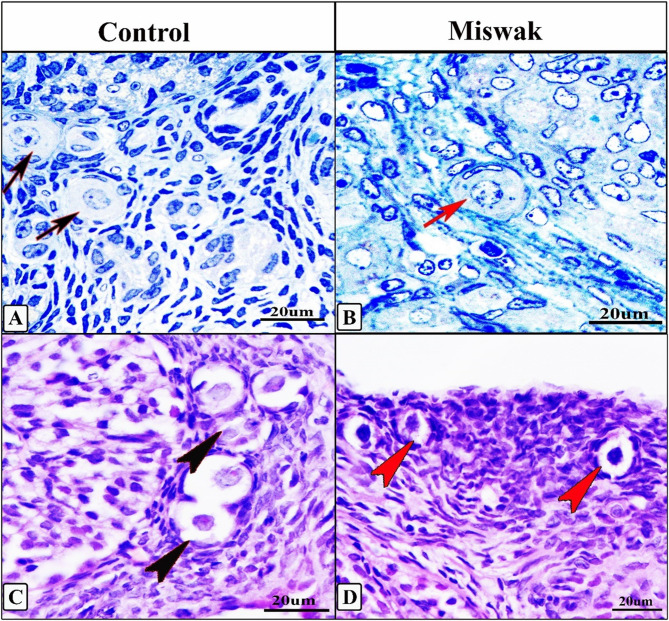
Semithin sections of ovarian cortex stained with toluidine blue (**A** and **B**) and paraffin sections of ovarian cortex stained by Hx and E (**C** and **D**) showing: **A**: Normal primordial follicles (black arrow) in the ovarian cortex of CG. **B**: Normal primordial follicle (red arrow) in the ovarian cortex of MTG. **C**: Polyoocyte follicle (black arrowhead) in ovarian cortex from CG. **D**: Normal primordial follicle (red arrow) in the ovarian cortex from MTG. Scale bar: 20 μm

**Fig. 5:**
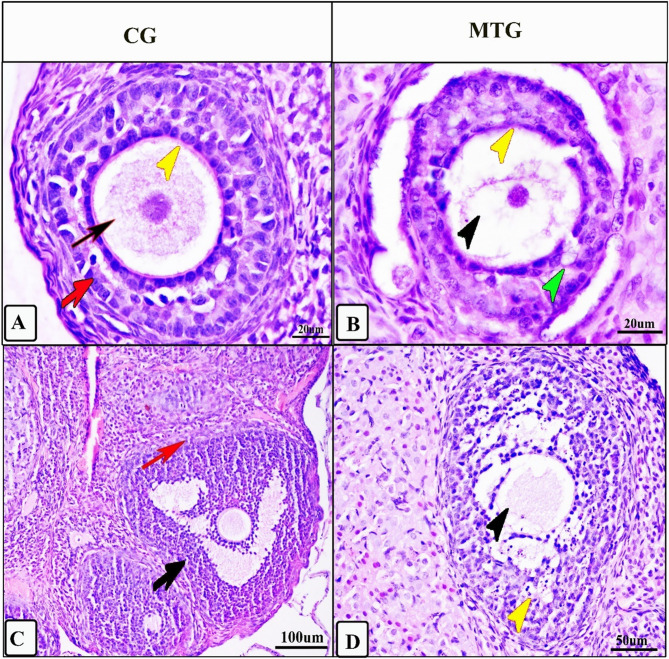
Paraffin sections of ovarian follicles stained by Hx and E displaying: **A**: Normal secondary follicle with clear homogenous acidophilic zona pellucida (yellow arrowhead), zona granulosa (red arrow) and oocyte (black arrow) from CG. **B**: Atretic follicle with disintegration of oocyte (black arrowhead) and granulosa cells (green arrowhead) and thinning of zona pellucida (yellow arrowhead) in the MTG ovary. **C**: Normal early antral follicle with normal zona granulosa (black arrow) and theca folliculi (red arrow) in ovary from CG. **D**: Atretic follicle in ovary from MTG showing apoptosis in the oocyte (black arrowhead) and in the granulosa cells (yellow arrowhead). Scale bar (A and B): 20 μm, C: 100 μm, and D: 50 μm

The development of ovarian follicles began with primordial follicles. Each primordial follicle consisted of an oocyte (immature egg) surrounded by a single layer of flattened follicular cells, with no obvious difference between the CG and MTG. However, many polyoocyte follicles were observed in the CG (Fig. 4). The primordial follicles then developed into primary follicles, which were composed of an oocyte surrounded by the zona pellucida and a single layer of small cuboidal follicular cells (unilaminar primary follicles). In the secondary ovarian follicles of the CG, the follicular cells appeared as stratified cuboidal granulosa cells. Fluid-filled cavities emerged between the granulosa cells and subsequently coalesced to form the antrum in the antral follicles. Differences between the two groups became evident in the growing ovarian follicles, where primary, secondary, and early antral follicles exhibited structural characteristics of atresia in the MTG. Apoptosis of granulosa cells and oocytes was observed in the ovaries of the MTG (Fig. 5).

### The mature follicle

**Fig. 6:**
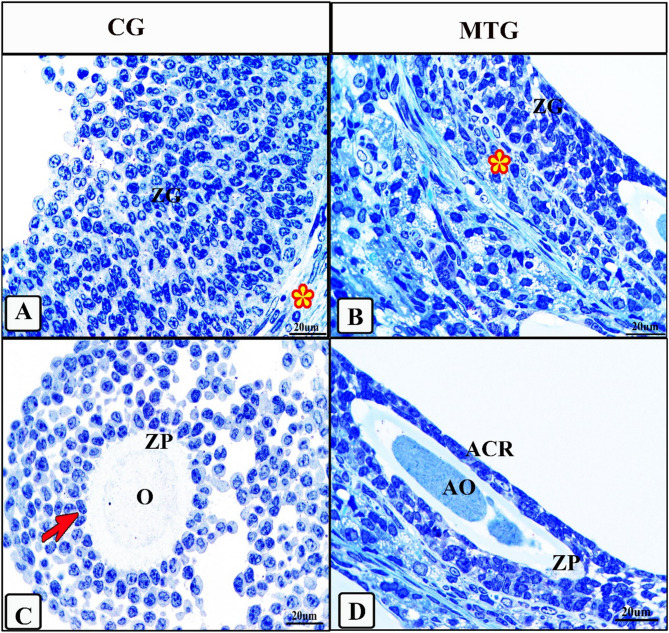
Semithin sections stained with toluidine blue from ovaries of CG and MTG. **A**: Normal zona granulosa (ZG) and normal theca folliculi (star) in CG. **B**: Disintegrated atretic granulosa cells (ZG) and hypertrophied theca interna (star) in ovary from MTG. **C**: Normal oocyte of mature follicle (O), zona pellucida (ZP) and corona radiate (arrow) in ovary from CG. **D**: Atretic oocyte (AO) in atretic follicle in ovary from MTG, with thick zona pellucida (ZP) and atretic corona radiata (ACR). Scale bar (A-D): 20 μm

**Fig. 7:**
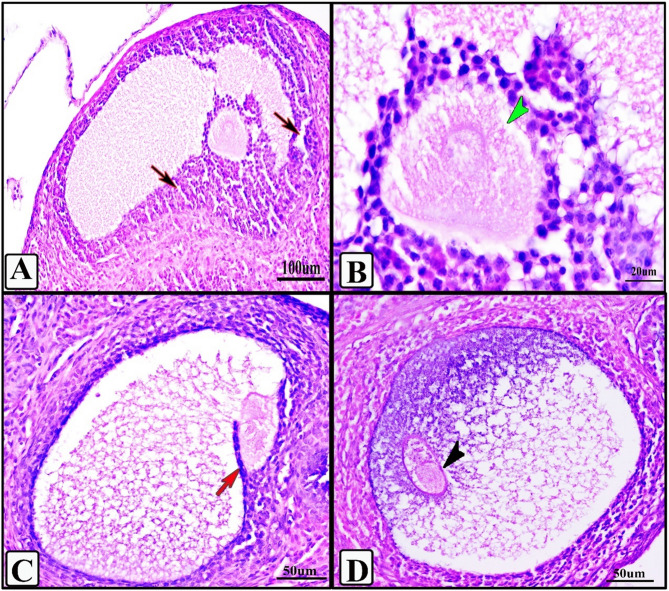
Paraffin sections of atretic mature follicles in ovaries from MTG stained by Hx and E displaying: **A**: Apoptosis and disintegration of granulosa cells (black arrow). **B**: Disintegration in zona pellucida (green arrowhead). **C**: Degeneration in corona radiata and cumulus oophorus (red arrow). **D**: Apoptotic oocyte floating in the antral cavity (black arrowhead). Scale bar A: 100 μm, B: 20 μm, and (C and D): 50 μm

By semithin and paraffin sections, the mature follicles in the CG displayed morphologically normal, intact structures. Each follicle consisted of an oocyte surrounded by a homogeneous zona pellucida and corona radiata cells. Stratified zona granulosa cells surrounded the follicular antrum and formed the cumulus oophorus. The follicular basement membrane and the surrounding theca folliculi appeared normally intact. In the MTG, mature follicles exhibited morphological characteristics of cellular apoptosis, indicating follicular atresia. They showed clumps of nuclear material, fragmented nuclei, and apoptotic bodies. Remarkable thickening and disintegration of the zona pellucida, degeneration of the corona radiata and cumulus oophorus, as well as vacuolation in the zona granulosa, were observed. A floating apoptotic oocyte appeared in the antrum (Figs. 6 and 7).

**Fig. 8:**
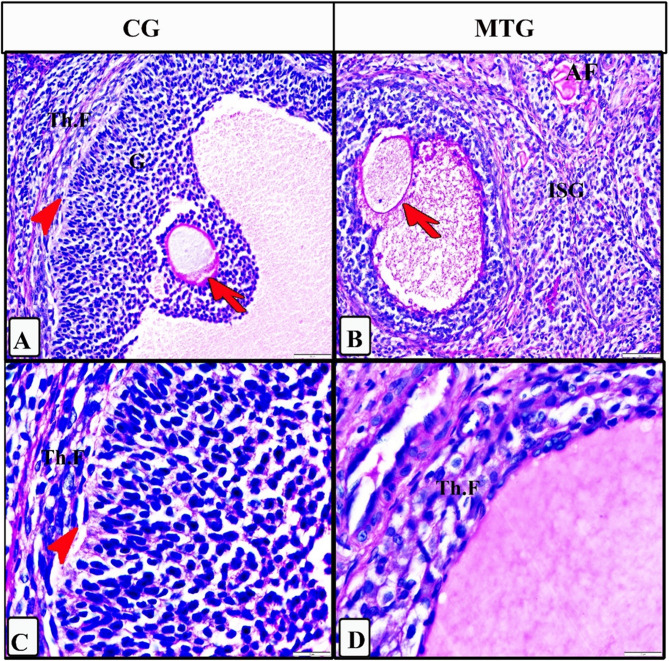
Paraffin sections stained by PAS and Hx showing structure of mature follicle in ovaries from CG and MTG: **A**: PAS-positive normal follicular zona pellucida (arrow) and follicular basement membrane (red arrowhead), theca folliculi (Th.F), granulosa cells (G) in non-treated CG. **B**: Thinning in zona pellucida (arrow) in atretic mature follicle in MTG. Note: The atretic follicle (AF) surrounded with hypertrophied theca folliculi (Th.F), interstitial gland (ISG). **C**: Structurally continuous basement membrane (red arrowhead) and normal theca folliculi (Th.F) can be seen in CG. **D**: Hypertrophy of theca folliculi (Th.F) and disappearance of the basement membrane are observed in MTG. Scale bar (A and B): 50 μm, and (C and D): 20 μm

Paraffin sections stained with PAS and Hx clearly showed the effects of MAE administration on the structure of mature ovarian follicles. In the MTG, the morphological features of mature follicles were difficult to distinguish. The mature follicles exhibited signs of follicular atresia, including thinning of the zona pellucida, as well as hypertrophy and proliferation of theca interna cells. Additionally, the basement membrane of the zona granulosa could not be clearly differentiated. In contrast, in the CG, the structural layers of mature follicles appeared normal, with a PAS-positive zona pellucida and a continuous basement membrane (Fig. 8).

**Fig. 9:**
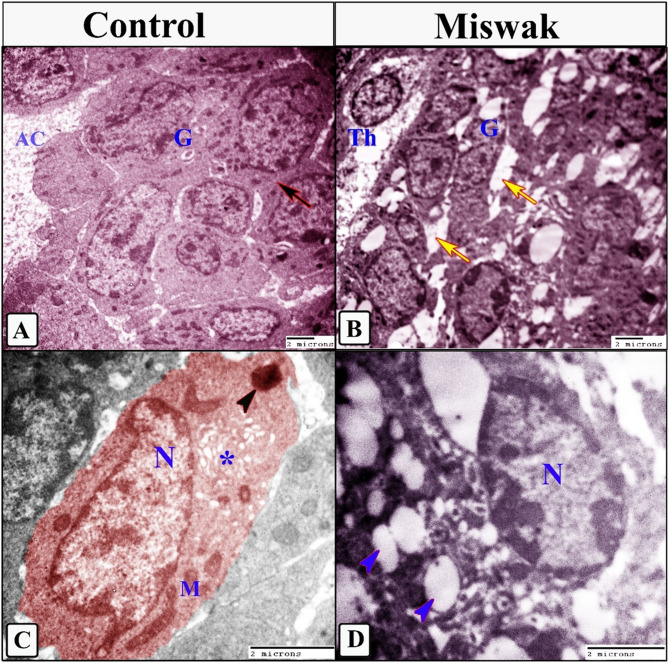
TEM -micrographs showing the differences between granulosa cells of mature follicles in CG and MTG. **A**: Stratified stacked granulosa cells (G) in CG showing antral cavity (AC). Notice narrow spaces between granulosa cells (black arrow). Mag. 3600x. **B**: Disintegrated granulosa cells (G) showing irregular clear intercellular spaces (yellow arrows) in MTG. Theca cell (Th.F) with vacuolated cytoplasm. Mag. 3600x. **C**: Higher magnification of granulosa cells from mature follicles in CG showing large nucleus (N), well developed SER (star), abundant mitochondria (M), and lysosome with heterogenous content (black arrowhead). Mag. 10000x. **D**: Higher magnification of granulosa cells from atretic mature follicles in MTG showing vacuolated cytoplasm (red arrowhead). Scale bar (A-D): 2 μm

TEM revealed that the granulosa cells in the CG group were arranged in an orderly, normally stacked structure. Their cytoplasm contained abundant mitochondria of varying sizes, well-developed smooth endoplasmic reticulum, and some lipid droplets. On the other hand, the MTG group exhibited irregular, clear intercellular spaces within the granulosa layer and vacuolated cytoplasm (Fig. 9).

### Effect of MAE on caspase-3 expression (apoptosis)

**Fig. 10:**
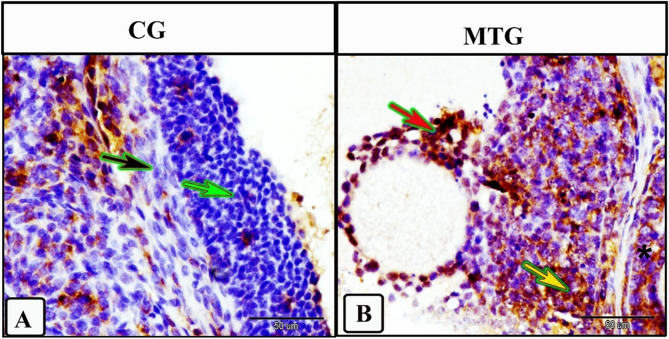
Paraffin sections of rat ovary stained by immunohistochemistry for detection of caspase-3 expression. Brown color indicates a positive expression, and blue indicates the counterstain. **A**: Low caspase-3 immunoexpression in the granulosa cells (green arrow) and theca interna cells (black arrow) of the normal antral follicles in CG. **B**: High caspase-3 immunoexpression in the granulosa cells (yellow arrow), corona radiata (red arrow) and theca interna cells (star) of the atretic antral follicles in MTG. Scale bar (A and B) = 50 μm

Immunohistochemical localization of caspase-3 in the CG showed low immunoexpression in the granulosa cells and theca interna cells of antral follicles. In contrast, the granulosa cells, corona radiata cells, and theca interna cells of atretic mature follicles in the ovary of the MTG showed high immunoexpression of caspase-3 (Fig. 10).

### Effect of MAE on the PR expression

**Fig. 11:**
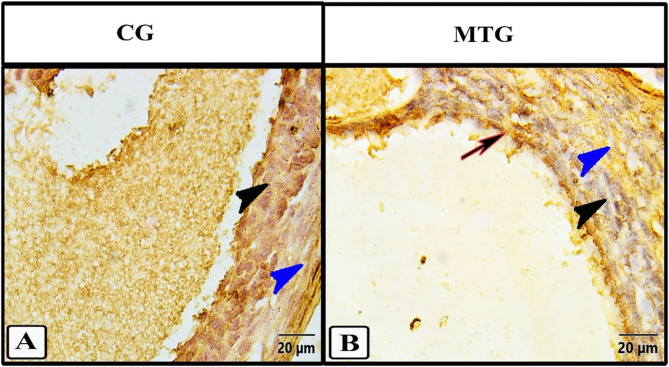
Paraffin sections showing the immunohistochemical localization of PR in the ovaries of rat. Brown color indicates a positive reaction. **A**: Strong positive PR immunoexpression in the granulosa cells (black arrowhead), and theca folliculi cells (blue arrowhead) of the antral follicle of CG. **B**: Mild PR immunoreactivity in the few remaining granulosa cells (black arrow), in the theca interna cells (black arrowhead), and theca externa cells (blue arrowhead) of the antral follicle of MTG. Scale bar (A and B) = 20 μm

By using the immunohistochemical technique, the ovaries of rats in CG showed strong positive immunoexpression for PR in the granulosa cells and theca folliculi cells of the antral follicles. But the ovaries in MTG showed mild PR immunoexpression in the remaining granulosa cells and in the theca folliculi cells of the atretic antral follicles (Fig. 11).

### Effect of the MAE on ERA expression

**Fig. 12:**
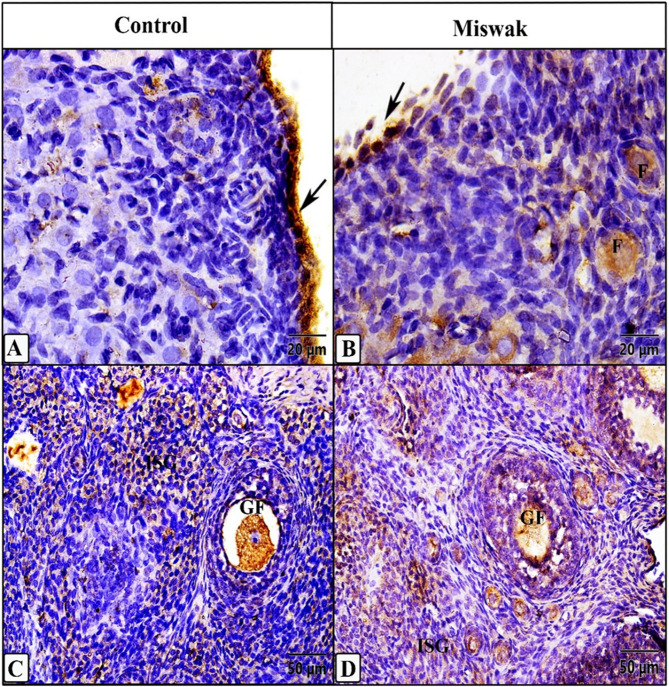
Paraffin sections showing the immunohistochemical localization of ERA in the rat ovary. Brown color indicates a positive expression. **A**: CG showed a strong positive reaction in ovarian surface epithelium (black arrow). **B**: MTG showed strong positive reaction in ovarian surface epithelium (black arrow) and moderate expression in the oocyte and follicular cells of the primordial follicle (F). **C**: CG showed moderate expression in the oocyte and granulosa cells of the growing follicle (GF) and interstitial gland cells (ISG). **D**: MTG showed moderate expression in growing follicles (GF) and interstitial gland cells (ISG). Scale bar (A and B) = 20 μm, and (C and D) = 50 μm

**Fig. 13:**
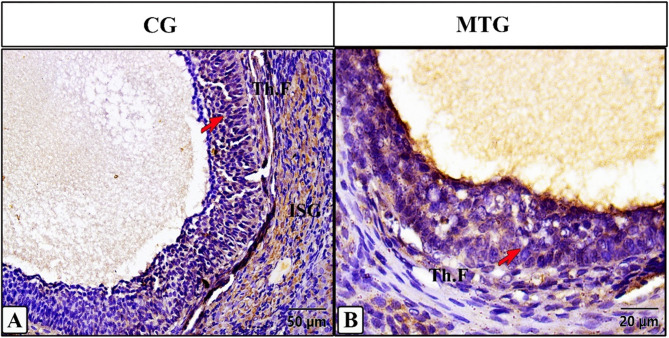
Paraffin sections showing the immunohistochemical localization of ERA in the rat ovary. Brown color indicates the positive reaction. **A**: CG showed moderate ERA immunoexpression in some granulosa cells (red arrow) and theca folliculi cells (Th.F) of antral follicle. Note the moderate ERA immunoexpression in the interstitial glands cells (ISG). **B**: MTG showed moderate ERA immunoexpression in some granulosa cells (red arrow) and theca folliculi cells (Th.F) of the early atretic antral follicle. Scale bar A = 50 μm, and B = 20 μm

Immunohistochemical localization of ERA in the rat ovary displayed strong ERA immunoexpression in the ovarian surface epithelial cells in both the CG and MTG groups. In addition, moderate ERA immunoexpression was observed in some granulosa cells of the growing follicles and in the interstitial gland cells in both groups (Fig. 12). In the CG group, mature follicles showed moderate ERA immunoexpression in some granulosa cells and theca folliculi cells. Similarly, early atretic antral follicles in the MTG group exhibited moderate ERA immunoexpression in some granulosa cells and theca folliculi cells (Fig. 13).

### Quantification of immunostaining

**Fig. 14:**
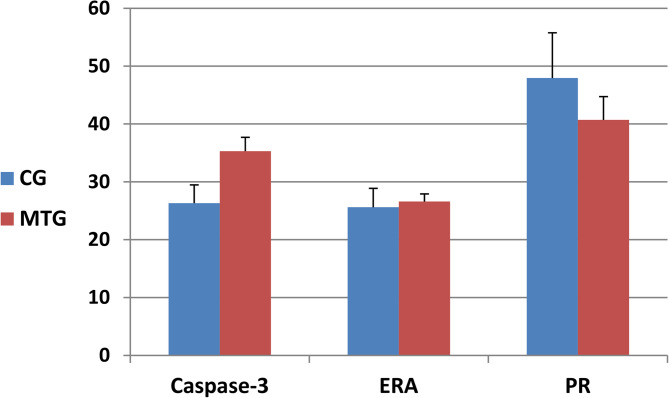
Histogram showing the effect of MAE administration on the immunoexpression of caspase-3, ERA, and PR in the rat ovary

Quantification of immunostaining markers revealed that in the MTG, the presence of caspase-3 immunostaining was significantly increased compared with the CG (35.29 ± 2.41 vs. 26.31 ± 3.16; *p* < 0.05). However, no significant differences were observed in ERA or PR immunoexpression between the two experimental groups (*p* > 0.05) (Table 3; Fig. 14).

**Table 3:** Showing the effect of MAE administration on the immunoexpression of caspase-3, ERA, and PR in the rat ovary

Groups	CG	MTG	*P* value
Caspase-3	26.31 ± 3.16^a^	35.29 ± 2.41^b^	0.047
ERA	25.60 ± 3.27^a^	26.59 ± 1.32^a^	0.793
PR	47.94 ± 7.82^a^	40.70 ± 4.04^a^	0.443

Values (Means ± SE) with different superscripts (a and b) in the same row are significantly different (*P* < 0.05) between the CG and MTG

### Morphometric measurements

#### Effect of the MAE on the number of different ovarian follicles

In this experiment, the mean number of primordial and growing follicles was not significantly different between the CG and MTG groups. However, the number of atretic antral follicles was significantly increased, and the number of mature follicles was significantly decreased in the MTG compared to the CG. Morphologically intact mature follicles were absent in the MTG (Table 4).

**Table 4:** Showing the effect of MAE administration on the mean number of different types of ovarian follicles in the MTG compared to the CG

Groups Follicles	CG	MTG
Primordial follicles	6.75 ± 1.4^a^	7.25 ± 4.3^a^
Growing follicles	6.25 ± 1.4^a^	4 ± 1.08^a^
Mature follicle*	1.750 ± 0.25^a^	0.00 ± 0.00^b^
Atretic antral follicles	0.5 ± 0.28^a^	4.5 ± 0.8^b^

The differences are non-significant except; the number of atretic antral follicles increased significantly, and the number of mature follicles decreased significantly in the MTG compared to the CG. Values (Means ± SE) with different superscripts (a and b) in the same row are significantly different (*P* < 0.05) between the CG and MTG groups. The numbers of primordial, growing, and atretic antral follicles were analyzed using the unpaired Student’s t-test

*In the mature follicle row, “0 ± 0” represents the absence of within-group variability, as no animals in that group possessed mature follicles. The numbers of mature follicles were analyzed using the Mann Whitney U test (non-parametric)

### Effect of the MAE on the weight of the rat ovary

In this experiment, ovarian weight was not significantly decreased in the MTG compared to the CG (Table 5).

**Table 5:** Showing the effect of MAE administration on the mean weight of the two ovaries in CG and MTG

Groups	CG	MTG
Weight of 2 ovaries (mg)	0.162 ± 0.009^a^	0.137 ± 0.013^a^

Values (Means ± SE) with the same superscripts in the same row are insignificantly different (*P* > 0.05) between CG and MTG groups

## Discussion

### Effect of MAE on estrous cycle

The examination of vaginal smears in the present study revealed that MTG animals exhibited a smear pattern consistent with the diestrus phase. Whereas most rats in the CG group showed smear patterns corresponding to the proestrus and estrus phases. The smears from acyclic animals with prolonged diestrus resembled those of normal cycling diestrus but contained more leukocytes or mucus [39]. Therefore, the administration of miswak may induce acyclic diestrus in female albino rats. This phase is characterized by the presence of polymorphonuclear leukocytes and epithelial cells in the vaginal smear.

### Effect of MAE on follicular development and atresia

Our results showed an increase in the number of atretic follicles in the MTG compared with normal follicular development in the control group. Similar results have been obtained with miswak leaf and stem extract, which trigger follicular atresia and exhibit anti-ovulatory effects in female albino rats [17]. Therefore, our observations suggest that MAE may delay ovulation by causing follicular atresia and exerting anti-ovulatory effects. Follicle survival factors that are suppressing follicular atresia are epidermal growth factor (EGF), transforming growth factor alpha (TGFa), basic fibroblast growth factor (bFGF), insulin-like growth factor-1 (IGF-1), and estrogens, while androgens and interleukin-6 (IL-6) are factors enhancing apoptosis [7]. Atresia from primordial to antral follicles is due to decreased growth and developmental factors, but atresia of large antral follicles and preovulatory follicles is due to decreased gonadotropins (FSH and LH) [3].

In the current study, caspase-3 immunohistochemistry showed high expression in the MTG. This expression was observed in granulosa cells and theca interna cells of atretic antral follicles. In contrast, expression was low in the CG. Specifically, most granulosa cells and theca interna cells of normal non-atretic follicles showed no caspase-3 expression. This means that MAE may suppress the follicle survival factors and enhance the follicle atretic factors. Caspase-3 belongs to a family of cysteine proteases. Its activation is important for apoptosis. Caspase-3 is activated in the apoptotic cells by both extrinsic (death ligand) and intrinsic (mitochondrial) pathways that produce programmed cell death. The active form of caspase-3 was localized in the granulosa cells of large preantral and small antral follicles, and in some oocytes of small preantral follicles [40]. The B-cell lymphoma 2 (Bcl-2) family is important in regulating the intrinsic initiation of apoptosis through mitochondrial pathways. Caspase-3-dependent cleavage of Bcl-2 appears to promote further caspase activation as part of a positive feedback loop; it localizes mitochondria and causes the release of cytochrome c into the cytosol [41]. It is suggested that MAE may affect growth and developmental factors and gonadotropin production and apoptotic proteins as Caspase-3 expression which all regulates follicular atresia.

### Effect of MAE on ERA expression

In the present study, ERA immunoexpression was observed in ovarian surface epithelium, follicular cells of primordial, growing, and mature follicles, as well as interstitial gland cells and theca cells of both groups. This cell-specific ERA distribution aligns with previous reports in the human [42] and rat ovary [43] where ERA was localized in theca cells, interstitial gland cells, and germinal epithelial cells. Although estrogen receptor beta (ERB) is the predominant form of ER in the granulosa cells of small, growing, and preovulatory follicles, conversely, ERA is more abundant in the interstitium and theca cells [43–45]. However, granulosa cells of atretic follicle showed only weak or no staining to (ERB) [43].

### Effect of MAE on PR expression

In the control group, PR immunoexpression revealed strong positive reactions in granulosa cells, cumulus oophorus, corona radiata, and theca folliculi. In MTG, mild or weak immunoexpression was observed in granulosa cells and corona radiata, while strong immunoexpression was present in theca folliculi of atretic follicle. Progesterone acts via classical nuclear receptor to regulate apoptosis in granulosa cells of preovulatory follicles [46]. The PR is temporarily expressed in granulosa cells by LH surge in rodents [47, 48]. Animals in proestrous and in the preovulatory stage had transient high progesterone levels [49]. The expression of PR mRNA was transient and was tightly coupled to the preovulatory LH surge on proestrous evening. PR mRNA was localized to the granulosa cells of mature ovarian follicles during the estrous cycle. In cycling animals treated with pentobarbital to block the preovulatory LH surge, no induction of PR mRNA on proestrous evening was observed. This transient, hormonally regulated, and cell-specific expression of the PR gene in the rat ovary strongly suggests an important intraovarian function for progesterone during the rat reproductive cycle [47, 48].

### Can miswak be used as a contraceptive?

Our results about effect of MAE administration on the ovarian follicles of the female albino rats are consistent with those reported for hormonal contraceptives that cause decrease in the ovarian size, and follicular growth and suppression of the ovulation [20]. Contraceptives such as Noristerat (NET-EN) cause follicular atresia and degeneration of growing follicles and with higher doses cause arrest of endometrial growth. Depo-Provera (DMPA) at high doses results in blocking ovulation [21]. The use of Norethisterone Acetate (NETA) has negative effects on ovarian follicles, including a reduction in their number and signs of atresia across all stages [50].

### Effect of other phytoestrogen plants on ovarian function

Phytoestrogens are naturally occurring plant compounds that are structurally and functionally like 17β-estradiol and have biological value [16]. Phytoestrogenic plants contained saponins and flavonoids, which can increase the level of circulating estrogen in the female rats. The elevated level of estrogen interferes with fertility [51]. It was found that rats treated with 500 mg/kg body weight of *Cleome gynandra* showed a decrease in the number of primordial and growing follicles with an increase in the number of atretic follicles, especially mature ones [52].

### Effect of MAE on hormonal level

Assessment of the influence of MAE on blood hormonal level showed a non-significant decrease in FSH, LH and estrogen hormones. A continuous downward trend was noted. However, these results should be interpreted with caution, as lack of statistical significance precludes any definitive statement regarding a direct inhibitory action on the hypothalamic-pituitary-ovarian (HPO) axis. It is biologically plausible that some slight non-significant decreases in these hormones could impact follicular development, but the present data do not support this mechanism on a statistical basis.

Phytochemical analysis of *S. persica* has identified compounds with potential estrogenic or endocrine-modulating properties, including flavonoids and sterols [12–15], but it is unknown whether these compounds contributed to the hormonal trends observed. FSH and LH play an essential role in follicular recruitment, development, and ovulation. Therefore, even small reductions in their levels may explain the major drop in mature follicles observed in this study. Nonetheless, because the hormonal changes were not statistically significant, such interpretations remain speculative. Follicle survival during the normal reproductive cycle depends on adequate FSH support during critical developmental stages; follicles receiving insufficient FSH are prone to atresia [3]. Likewise, LH is important for early follicle development and increasing estrogen production, which supports oocyte maturation [53]. Also, LH acts on preovulatory granulosa cells to regulate specific genes necessary for ovulation and luteinization [54]. A possible, though not statistically confirmed, explanation for the observed decrease in blood estrogen levels following miswak treatment is the increase in antral follicular atresia. This was because the granulosa cells are the main source of estrogen secretion [55]. However, this suggested connection should be hypothesis-generating rather than definitive because estrogen reduction was not significant.

It was found that the administration of *Salvadora persica* aqueous extract at a dose of 7 mg/100 g body weight for 30 days reduced estrogen and increased progesterone levels [18]. Other research found that the aqueous and alcoholic *Salvadora persica* roots extract at concentrations (250 and 500) mg/kg of body weight in female albino rats exhibited antifertility activity. Both extracts resulted in a significant decrease in the level of FSH and LH and a significant increase in the level of estrogen hormone compared to the control group [56]. This difference in the level of estrogen hormone from our finding may be due to the difference in the dose, duration of treatment, and type of extract. This trend, although not significant, is consistent with the morphological finding of reduced follicular growth, but our sample size or treatment period may have limited the detection of significant hormonal changes. Future studies with larger sample size and longer treatment durations are necessary to assess whether modulation of the HPO axis is the predominant mechanism of action for the observed effects of *S. persica* on ovarian folliculogenesis. Since the differences in FSH, LH, estrogen and receptor-related measures were not statistically significant; any mechanistic interpretation involving HPO axis, estrogen reduction or receptor-mediated pathways should be made with caution, although the trend in hormones corresponds to the follicular changes.

### Limitations of the study

The limitations of our study include the relatively small sample size per experimental group, and a lack of molecular investigations. Furthermore, this preliminary study did not evaluate the systemic safety aspects of the extract nor establish a dose response relationship. Future studies addressing these limitations are necessary to confirm and expand upon these promising initial observations. Future molecular studies should focus on essential regulatory pathways of folliculogenesis, including the PI3K/AKT/mTOR/FOXO3 signaling axis (vital for follicle activation and growth), the TGF-β/SMAD pathway (associated with granulosa cell proliferation and differentiation), as well as the expression of growth factors (GDP9, BMP15) to clarify the specific mechanism of *S. persica* extract on ovarian follicle dynamics.

## Conclusion

Our findings suggest that MAE can inhibit follicular development and induce follicular atresia, indicating a potential antifollicular and/or antiovulatory effect. However, these findings are preliminary and require confirmation through dose-response, fertility, toxicity, and clinical studies.

## Data Availability

The datasets used and/or analysed during the current study are available from the corresponding author on reasonable request.

## Abbreviations

Bax: Bcl-2 Associated X-protein
BCl-2: B-cell lymphoma 2
bFGF: Basic fibroblast growth factor
CG: Control group
EGF: Epidermal growth factor
ER: Estrogen receptors
ERA: Estrogen receptors alpha
ERB: Estrogen receptors beta
FSH: Follicle stimulating hormone
Hx and E: Hematoxylin and eosin
IGF-1: Insulin-like growth factor-1
IL-6: Interleukin-6
LH: Luteinizing hormone
MAE: Miswak aqueous extract
MTG: Miswak treated group
PAS: Periodic acid-Schiff’s
PBS: Phosphate- buffered saline
PR: Progesterone receptor
TEM: Transmission electron microscope
TGFa: Transforming growth factor alpha
HPO: Hypothalamic-pituitary-ovarian axis
